# The Role of Interferon Regulatory Factor 1 in Regulating Microglial Activation and Retinal Inflammation

**DOI:** 10.3390/ijms232314664

**Published:** 2022-11-24

**Authors:** Xu Yang, Valeria Diaz, Hu Huang

**Affiliations:** 1 Department of Ophthalmology, School of Medicine, University of Missouri, Columbia, MO 65212, USA; 2 Aier Eye Hospital Group, Aier Eye Institute, Changsha 410015, China

**Keywords:** CRISPR-Cas9, IRF1, microglia, retina inflammation, transcription factor

## Abstract

Microglia are resident immune cells in the central nervous system (CNS). Microglial activation plays a prominent role in neuroinflammation and CNS diseases. However, the underlying mechanisms of microglial activation are not well understood. Here, we report that the transcription factor interferon regulatory factor 1 (IRF1) plays critical roles in microglial activation and retinal inflammation by regulating pro- and anti-inflammatory gene expression. IRF1 expression was upregulated in activated retinal microglia compared to those at the steady state. IRF1 knockout (KO) in BV2 microglia cells (BV2^ΔIRF1^) created by CRISPR/Cas9 genome-editing technique causes decreased microglia proliferation, migration, and phagocytosis. IRF1-KO decreased pro-inflammatory M1 marker gene expression induced by lipopolysaccharides (LPS), such as IL-6, COX-2, and CCL5, but increased anti-inflammatory M2 marker gene expression by IL-4/13, such as Arg-1, CD206, and TGF-β. Compared to the wild-type cells, microglial-conditioned media (MCM) of activated BV2^ΔIRF1^ cell cultures reduced toxicity or death to several retinal cells, including mouse cone photoreceptor-like 661 W cells, rat retinal neuron precursor R28 cells, and human ARPE-19 cells. IRF1 knockdown by siRNA alleviated microglial activation and retinal inflammation induced by LPS in mice. Together, the findings suggest that IRF1 plays a vital role in regulating microglial activation and retinal inflammation and, therefore, may be targeted for treating inflammatory and degenerative retinal diseases.

## 1. Introduction

Microglia, the primary resident macrophage-like immune cells in the central nervous system (CNS), play prominent roles during CNS development, hemostasis, and diseases [1,2]. For example, they are involved in establishing neuronal networks and contribute to remodeling synaptic connections via phagocyting apoptotic neurons and synapses in the developing brain. They surveil local microenvironments and maintain tissue homeostasis through their dynamic processes in the mature CNS. Conversely, they can promote pathogenesis by generating reactive species and inflammatory cytokines in varying neuronal degenerative and inflammatory diseases, such as Alzheimer’s disease [3], retinal degeneration [4], glaucoma, diabetic retinopathy (DR) [5,6], and age-related macular degeneration (AMD) [7]. The homeostatic and pathogenic roles of microglia in the CNS are attributed to their highly context-dependent heterogeneous features [8,9]. This phenomenon is manifested by their discrete morphological appearance (e.g., ramified vs. ameboid) and molecular signatures, such as sensome genes and inflammatory cytokines [10,11]. High-resolution single-cell RNA-sequencing (scRNA-Seq) technology reveals multiple retinal microglial cell subclasses or subpopulations based on their unique gene expressions [12,13]. However, how the CNS microglial heterogeneity correlates with their functional diversity remains unclear. 

Transcriptional factors (TFs) dictate the microglial cell identity and activation/polarization states in health and disease by controlling transcription of target genes. Based on the functional roles, TFs can be divided into three classes: general TFs (i.e., SP1, GABP, and YY1), lineage-determining TFs (LDTF, e.g., PU.1, RUNX, and IRF8), and signal-dependent TFs (SDTF), such as NFkB, AP-1, and LXR/RXR [14]. They interact with the promotor and enhancer DNA sequence elements of target genes and work hierarchically and cooperatively to determine microglia-specific gene expression programs. The transcriptome primarily defined by local microenvironments guides the microglial linage from erythromyeloid precursors (EMPs) in the yolk sac stage, the homeostatic state in developing and adult CNS, and the reactivation state associated with diseases. The synergistic interactions of microenvironmental cues, TFs, and DNA elements drive microglial phenotypic and functional diversity in response to the development, injury, stress, inflammation, and disease [8].

Interferon regulatory factors (IRFs) are the transcription factors induced in response to interferon (IFN), lipopolysaccharide (LPS), and other stimuli. Nine IRF family members (IRF1-9) have been identified and characterized [15,16,17]. Two additional orthologues named IRF10 and IRF11 have recently been discovered in fish and bird species [18]. These IRF family members share the conserved N-terminal DNA binding domain (DBD) and C-terminal IRF-association domain (IAD). They play critical roles in innate immune responses by regulating gene expression of the interferon and Toll-like receptor (TLR) signaling pathways. IRFs initiate gene expression by forming homo and/or heterodimers with IRF family members and/or other TFs, such as PU.1, STAT1, and NF-kB, which further bind with the interferon-sensitive response elements (ISRE), in which the GAAA motif is conserved. The genes regulated by IRFs are implicated in the pathophysiological functions of various diseases, such as bacterial and viral infections, cancer, immune, and inflammatory diseases. Copious studies have revealed the significant roles of IRF1 in the innate immune response under pathological conditions and diseases. For example, IRF1 is vital to mediate microglia-mediated neuroinflammation. IRF1 was required for IL-1β expression mediated by IRF8 in reactive microglia [19]. IRF1, LXRβ, and CEBPα regulated disease-associated microglia (DAM) gene expression (e.g., Apoe, Axl, and, Trem2) [20]. IRF1 was involved in vascular dysfunction related to DR by regulating the glial cell-specific expression of Sema4D and PlexinB1 [21]. IRF1 mediates inflammatory gene expression induced by LPS and anti-inflammatory effects of the BET inhibitor in human microglia HMC3 cells [22]. In the current study, we found that IRF1 regulates microglial polarization by promoting inflammation but inhibiting anti-inflammation gene expression and that IRF1 regulates the paracrine mechanisms from microglia to retinal cells, causing retinal cell death and retinal inflammation. 

## 2. Results

### 2.1. Increased IRF1 Protein Expression Levels in Activated Retinal Microglia 

**Figure 1:**
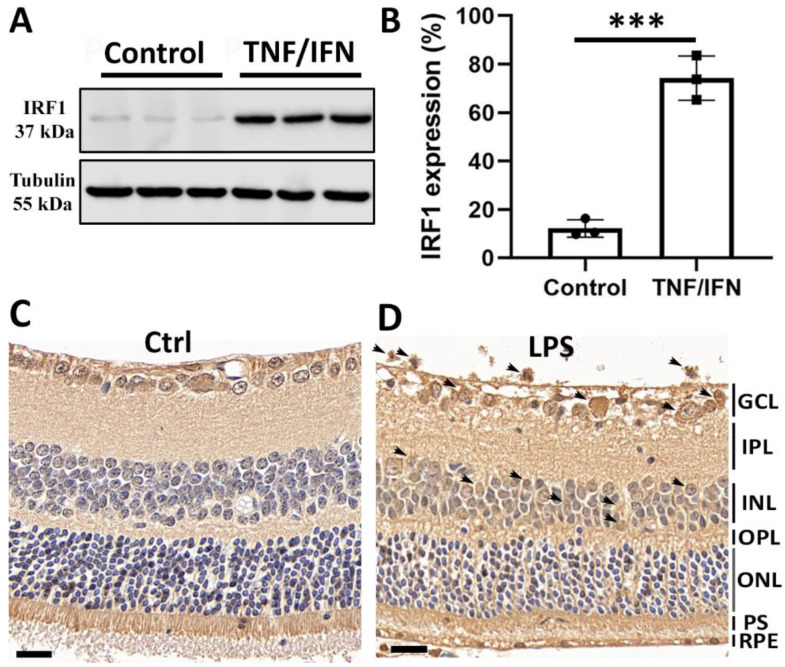
Increased IRF1 protein expression level in activated retinal microglia. (**A**,**B**) IRF1 protein upregulation in primary retinal microglia by 20 ng/mL tumor necrosis factor-alpha (TNFα) and 20 ng/mL interferon-gamma (IFNγ) for 24 h. The Western blot images of IRF1 ((**A**), Tubulin as loading control) and densitometric quantification results by ImageJ software ((**B**), % of Tubulin) are shown. Primary retinal microglia were derived from mice (~p10). The replicate numbers = 3. *** *p* < 0.001. (**C**,**D**) The increased IRF1 protein level in the mouse retina by lipopolysaccharide (LPS, 100 ng/µL) for 24 h. The representative IHC-DAB staining images (n = 3) for the PBS control (**C**) and LPS (**D**) treatments ae shown. Arrows indicate the IRF1 staining signals. Scale bar: 20 µm.

We recently reported that IRF1 mRNA transcripts were upregulated in retinal microglia activated by inflammatory cytokine (TNFα/INFγ) treatment compared with the non-stimulated microglia in primary microglial cell cultures using RNA-sequencing transcriptome and qPCR analyses [10]. Here, we further examined whether the IRF1 protein was upregulated in activated retinal microglia. To address this question, primary retinal microglia were cultured and treated with TNFα + INFγ (20 ng/mL each) for 24 h. The WB results showed that the IRF1 protein level was markedly increased in retinal microglia by TNFα/INFγ treatment (Figure 1A,B). Furthermore, immunohistochemistry (IHC) results demonstrated IRF1 upregulation in the retinal microglia of mice treated with LPS (Figure 1C,D).

### 2.2. IRF1 Knockout (KO) Defers BV2 Microglial Proliferation 

**Figure 2:**
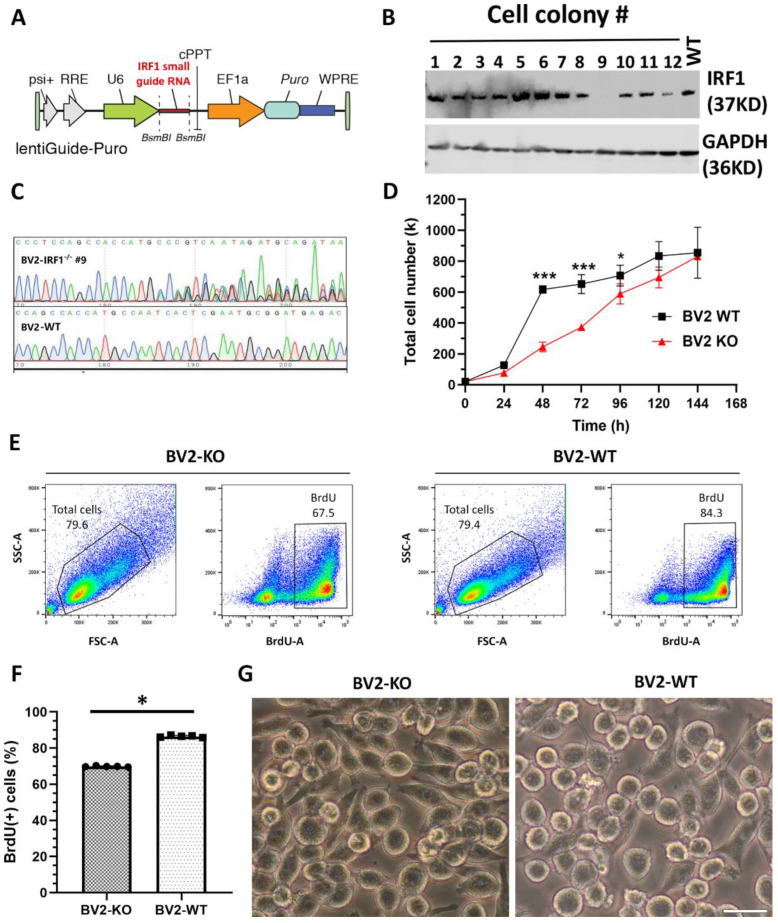
IRF1 knockout (KO) defers BV2 microglial proliferation. (**A**–**C**) IRF1 KO from BV2 microglial cells with CRISPR-Cas9 gene-editing technique. (**A**) The diagram depicting the insertion of IRF1 small guide (sg) RNA in the lentiviral plasmid. (**B**) The WB results showing that single-cell colony #9 is absent of IRF1 protein compared with the other cell colonies and wild-type (WT) cells. (**C**) DNA sequence showing the mutation of cell colony #9′s gene loci compared to the BV2-WT gene loci. (**D**–**F**) IRF1-KO defers the microglial cell proliferation rate. (**D**) Cell counts during BV2 cell growth. (**E**) Flow cytometry shows total live BV2 cells (left in the enclosed areas) and BrdU (+) BV2 cells (right in the boxed areas) for IRF1-KO BV2 (BV2-KO) and BV2-WT counterparts. (**F**) BrdU (+) cell numbers (%). n = 6. * *p* < 0.05; *** *p* < 0.001. (**G**) Cell morphological differences of BV2-KO and BV2-WT cells. Scale bar: 20 µm.

To elucidate the functional role of IRF1 in microglial homeostasis and activation, we deleted the IRF1 gene in BV2 microglia using the CRISPR-Cas9 genome-editing technique. IRF1 small guide RNA (sgRNA) was ligated into the LentiCRISPR2 plasmid to construct the targeting vector for gene-editing experiments (Figure 2A). Lentiviral particles were propagated and assembled via HEK293T cells. The assembled lentiviral particles were transfected into BV2 microglial cells; the antibiotic puromycin selected the transfected cells, and then the cells were diluted into a single cell for cultivation. The single-cell colonies were examined for KO efficiency with WB analysis. The results showed one single-cell colony (#9) had the most effective IRF1 protein deletion compared with other colonies and the WT cells (Figure 2B). The DNA sequencing result further confirmed that IRF1 gene loci of the single-cell colony were mutated compared with the wild type loci (Figure 2C). Therefore, this single-cell colony was assigned as IRF1-KO BV2 (BV2^ΔIRF1^) and used for phenotypical and functional examinations. 

The impact of IRF1-KO on microglial cell proliferation was first examined. The total cell numbers were statistically (*p* < 0.05) less in the BV2^ΔIRF1^ cells than in the WT controls during the cell growth phase (Figure 2D). BrdU incorporation and flow cytometry analyses revealed that the BV2^ΔIRF1^ cell cultures had ~68% BrdU (+) cells and that the BV2-WT cell cultures contained ~84% BrdU (+) cells (Figure 2E,F). It was also noted that the BV2^ΔIRF1^ cultures had more elongated (or less round) cells than those WT cells, suggesting the quiescent state of IRF1^-/-^ microglia relative to the WT cells (Figure 2G).

### 2.3. IRF1′s Involvement in Microglial Migration

**Figure 3:**
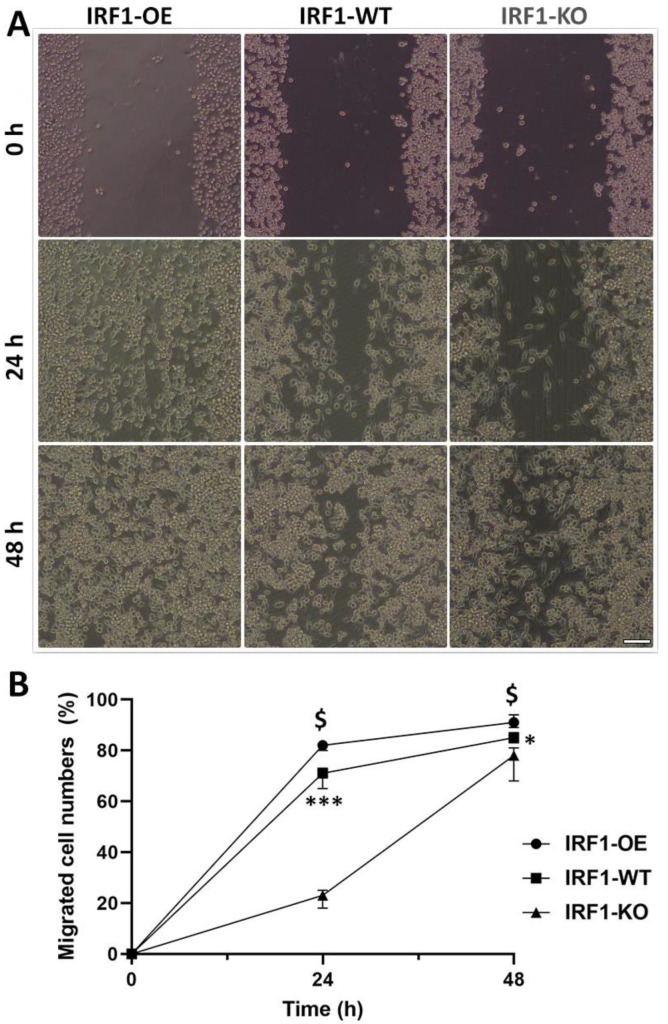
Microglial migration competency by cell scratch assay. The BV2 cells with the genotypes of IRF1-WT, IRF1-KO (BV2^ΔIRF1^), and IRF1-OE (overexpression) were cultivated to confluency. The cells were physically removed by scratching, and then the cell images were taken by differential interference contrast (DIC) microscopy at 24 and 48 h (**A**). 40× magnification. The cells in the scratched areas were quantified (**B**). N = 3; * *p* < 0.05; *** *p* < 0.001 (IRF1-WT vs. IRF1-KO). $ *p* < 0.05 (IRF1-OE vs. IRF1-WT).

A cell scratch assay was performed to examine whether IRF1 is involved in microglial migration. A straight line scratch was created on the confluent IRF1-WT, IRF1-KO, and IRF1-OE BV2 cells, and the cells were physically removed at 0 h. At 24 and 36 h, the cells migrated to the scratch/wounded area were counted and quantified. The migrated cell numbers were significantly less in the IRF1-KO BV2 (BV2^ΔIRF1^) than in the WT control cells at both time points. Conversely, the migrated cells were greater in the IRF1-OE BV2 cells than the WT counterparts (Figure 3). The results indicate that IRF1 is involved in microglial migration.

### 2.4. IRF1′s Role in the Phagocytosis of Activated Microglia

**Figure 4:**
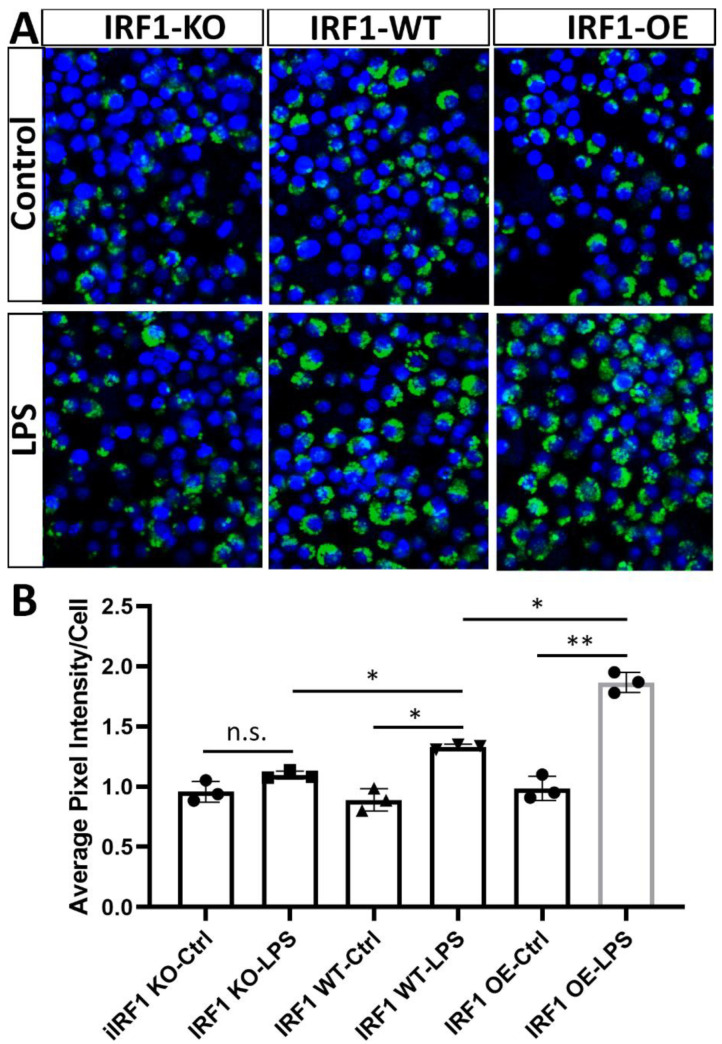
Phagocytosis evaluation of reactive microglia by Zymosan Bioparticles. Eighty percent confluent IRF1-KO, IRF1-WT, and IRF1-OE BV2 cells were treated with LPS (100 ng/mL) and PBS control for 24h. pHrodo^TM^ Green Zymosan Bioparticles (0.5 mg/mL) were added and incubated for 1.5 h. After PBS wash, the cells were fixed for imaging with confocal microscopy ((**A**), DAPI stained the nuclei), and the pixel intensity was analyzed with ImageJ software ((**B**), as described in Methods). The results are expressed as the mean pixel intensity per cell. 200× magnification. n = 3. n.s. *p* > 0.05; * *p* < 0.05; ** *p* < 0.01.

IRF1′s role in microglial phagocytosis was evaluated with the uptake of Zymosan bioparticles (pHrodo^TM^ Green) [23]. At the steady state (without stimulation), the Zymosan bioparticle levels were not significantly different between the microglial cells with IRF1-KO, -WT, and -OE. However, under LPS simulation conditions, IRF-KO microglia phagocytized less Zymosan bioparticles than WT cells; conversely, the microglia overexpressing IRF1 had more compared with the WT control cells (Figure 4A). The results revealed that BV2^ΔIRF1^ phagocytic competency is the same as that of the WT cells under non-stimulation conditions but is attenuated in the IRF1-KO microglia under LPS stimulation conditions, suggesting that IRF1 plays a role in microglial phagocytosis in response to inflammatory stimulation.

### 2.5. IRF1-KO Alters the Gene Expression Profile of M1 vs. M2 Activation State

**Figure 5:**
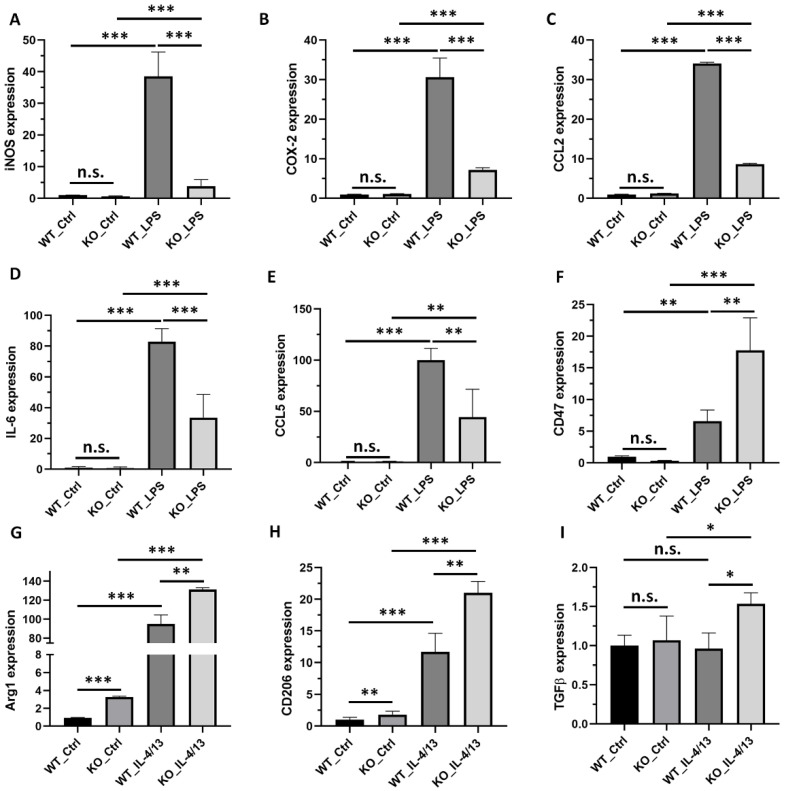
IRF1 KO alters gene expression of the M1/M2 microglial activation state. Eighty percent confluent IRF1-KO BV2 cells (BV2^ΔIRF1^) and wild-type (WT) cell cultures were treated with LPS or IL4/13 (PBS as control) for 24 h. The qPCR results for mRNA expression of iNOS (**A**), COX-2 (**B**), CCL2 (**C**), IL-6 (**D**), CCL5 (**E**), CD47 (**F**), Arg1 (**G**), CD206 (**H**), and TGFβ (**I**). The replicate numbers = 6. n.s. *p* > 0.05; * *p* < 0.05; ** *p* < 0.01; *** *p* < 0.001.

The effects of IRF1-KO on pro-inflammatory cytokine and M1-state marker gene expression were examined for the microglia with and without stimulation of LPS or TNFα (for M1 polarization). LPS upregulated the expression levels of five pro-inflammatory cytokine and marker genes (*NOS2*, *COX2*, *CCL2*, *IL6*, and *CCL5*) in both BV2^ΔIRF1^ (KO) and BV2-WT cells compared with their respective non-stimulated cells. However, the increased levels were significantly lower in the LPS-stimulated KO cells than in the LPS-stimulated WT cells (Figure 5A–E). Similar results were obtained in the TNFα-polarized (M1) microglia (Figure S1). However, *CD47*, which acts as a “do-not-eat-me” signal, [24] had higher expression in the LPS-activated KO microglia than the WT microglia (Figure 5F). The expression levels of anti-inflammatory factors Arg1 and CD206 were upregulated in both the IL-4/13-polarized WT and KO M2 microglia compared with the non-stimulated control cells. Their increased levels are higher in the M2-polarized KO cells than in the M2-polarized WT cells. Additionally, IL-4/13 did not upregulate TGFβ gene expression in the WT background but significantly increased its expression level in the IRF1-KO microglia compared to the non-stimulated KO cells and the stimulated WT cells (Figure 5G–I). These results suggest that IRF1 regulates the microglial polarization of the M1 state vs. the M2 state in vitro. 

### 2.6. IRF1 KO Reduces Microglial Cytotoxicity to Retinal Cells in Cultures

**Figure 6:**
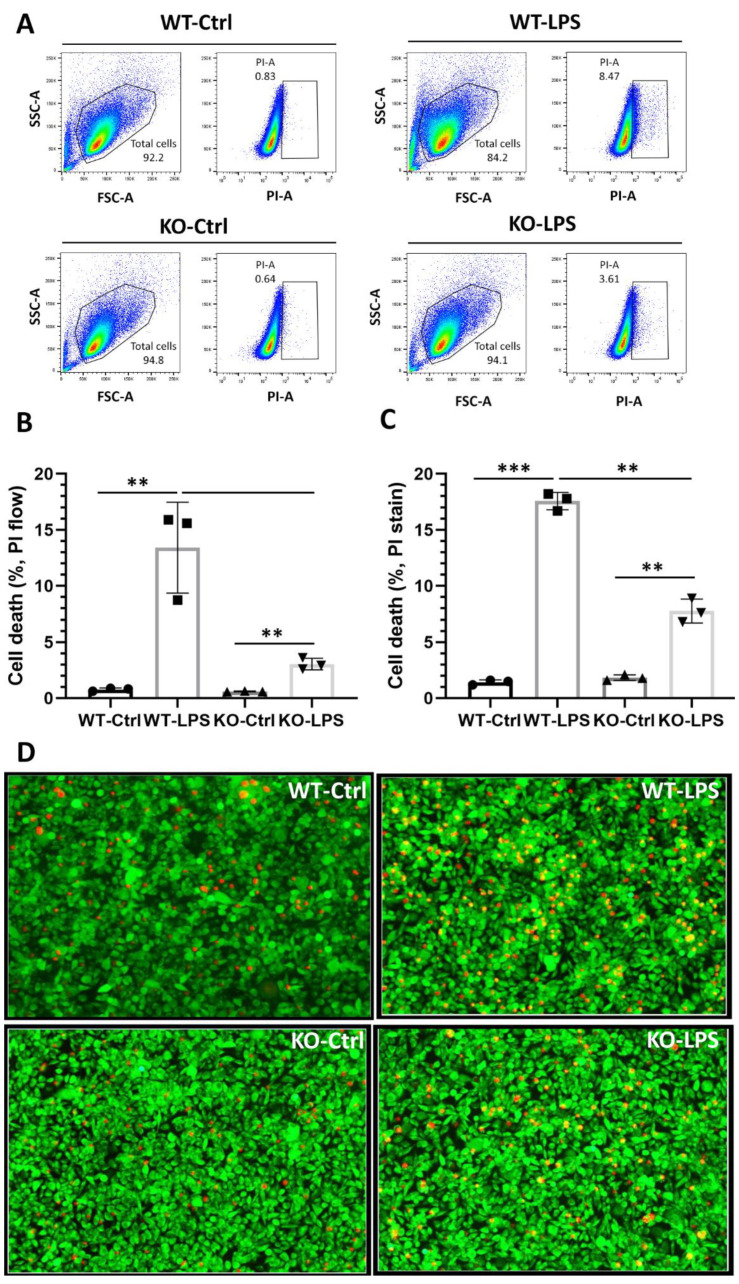
IRF1 deletion reduces microglial cytotoxicity to photoreceptors. (**A**) PI-gated flow cytometry analysis for the cone-like 661 W cells treated with PBS control (WT-Ctrl), the conditioned media from wild-type microglia with LPS (WT-LPS), the conditioned media from IRF1-KO microglia with PBS control (KO-Ctrl), and the conditioned media from IRF1-KO microglia with LPS (WT-LPS). Total live cells were shown in the enclosed areas of left panels. PI-positive dead cells were shown in the boxed areas in the right panels (**B**) Cell death quantification by flow cytometry analysis. (**C**) Cell death quantification of the calcium AM and PI stained cone-like 661 W cells images. (**D**) The representative images showing the Calcium AM (green) and PI (red) stained cone-like 661 W cells. 40× magnification. The replicate numbers = 3. ** *p* < 0.01; *** *p* < 0.001.

The involvement of IRF1 in regulating microglial paracrine effects on retinal cell viability and death was assessed. Microglia-conditioned media (MCM) from the IRF1-KO BV2 (*BV2*^ΔIRF1^) and the WT cells, which were activated with LPS (PBS control), were added to the cone photoreceptor-like 661 W cell cultures. Flow cytometry results revealed that the PI-positive dead cells are low in PBS control groups (~0.83% for WT cells and ~0.64 for IRF1-KO cells) and that the CM from the LPS-activated microglia-CM from both WT and KO groups largely induced 661 W cell death (Figure 6A,B). However, the dead cells (~3.6%) from IRF1-KO’s MCM are lower than those (~15.6%) from the WT conditions. The live/death staining by Calcium AM and PI also showed that the PI-positive cells from the activated WT microglia are greater in number than those from KO cells (Figure 6C,D).

Human ARPE19 cells and rat retinal neuron precursor R28 cells were further used to analyze paracrine effect from microglia to retinal cells regulated by IRF1. IRF1-KO also reduced microglial cytotoxicity to the two retinal cells, as shown by the increased PI-positive dead R28 cells and the decreased RPE ZO-1 protein levels (Figure S2).

### 2.7. IRF1 Knockdown Reduces Retinal Microglial Activation and Inflammation In Vivo

**Figure 7:**
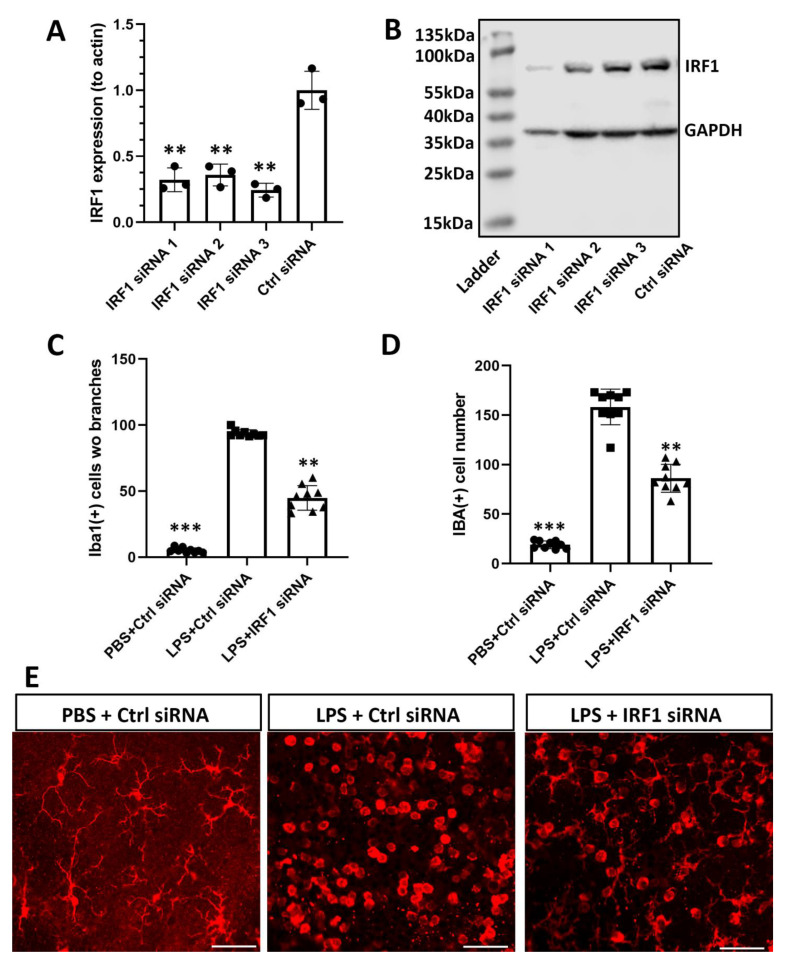
IRF1 knockdown reduces retinal microglia activation in vivo. (**A**,**B**) The LPS-activated BV2 cells were transfected with three IRF1 siRNAs and scrambled control siRNA (5 nM each). The IRF1 knockdown efficacy of siRNA at the mRNA level by qPCR (**A**) and at the protein level by Western blots (**B**). (**C**,**D**) The methylated IRF siRNA2 (1 nmol/µL, twice) was intravitreally injected into the mouse eyes at 1 and 4 days before LPS injection (100 ng/µL, 1 day) for microglial activation. Cell numbers without branches (**C**) and total Iba1 (+) cells (**D**) at the nerve fiber layer and ganglion cell layer (NFL/GCL) were quantified for the three groups: PBS + scrambled control siRNA, PBS + IRF1-siRNA, and LPS + IRF1-siRNA. (**E**) The representative Iba1 (+) cells were taken from the NFL/GCL of three groups. n = 9 areas of interest from 3 independent experiments and biological samples. ** *p* < 0.01; *** *p* < 0.001. scale bar = 20 µm.

Lastly, we investigated whether the IRF1 target could alleviate microglial activation and retinal inflammation in vivo. Three IRF1 siRNAs were designed and validated with BV2 microglial cells. As shown by qPCR results, three IRF1 siRNA had −80% knockdown efficiency at mRNA level compared with scrambled control siRNA (Figure 7A). WB results revealed various knockdown efficiencies at the protein level (Figure 7B). As siRNA2 reduced IRF1 protein expression by 60% without apparent off target effects as evaluated based on the GAPDH protein level, it was selected for the further in-vivo study. The chemically modified (methylated) IRF1-siRNA2 (with a longer half-life in vivo) was administrated into the mouse eye through intravitreal injection to knockdown IRF1. LPS was used to activate retinal microglia (PBS as control). In the scrambled siRNA + PBS control group, all Iba1 (+) microglia were ramified with multiple cellular processes indicating their “resting” states. By contrast, in the scrambled control siRNA + LPS group, no ramified microglia were observed; all microglia were activated and had no branches at the retinal nerve fiber layer and ganglion cell layer (NFL/GCL). However, IRF1-siRNA reduced LPS-activated microglia at NFL/GCL, as demonstrated by the decreased Iba1 (+) microglia number and the increased microglia with cellular processes compared with the control siRNA + LPS group. The results indicated that IRF1 knockdown by siRNA robustly inhibited microglia activation on the superficial retinal surface. It was noted the microglia located on the superficial retinal surface were mainly affected, but those on the intermediate and deeper retinal layers were affected minimally, likely due to the limited retinal penetration of siRNA at the low dose, short time, and negative charge (without carrier) used.

**Figure 8:**
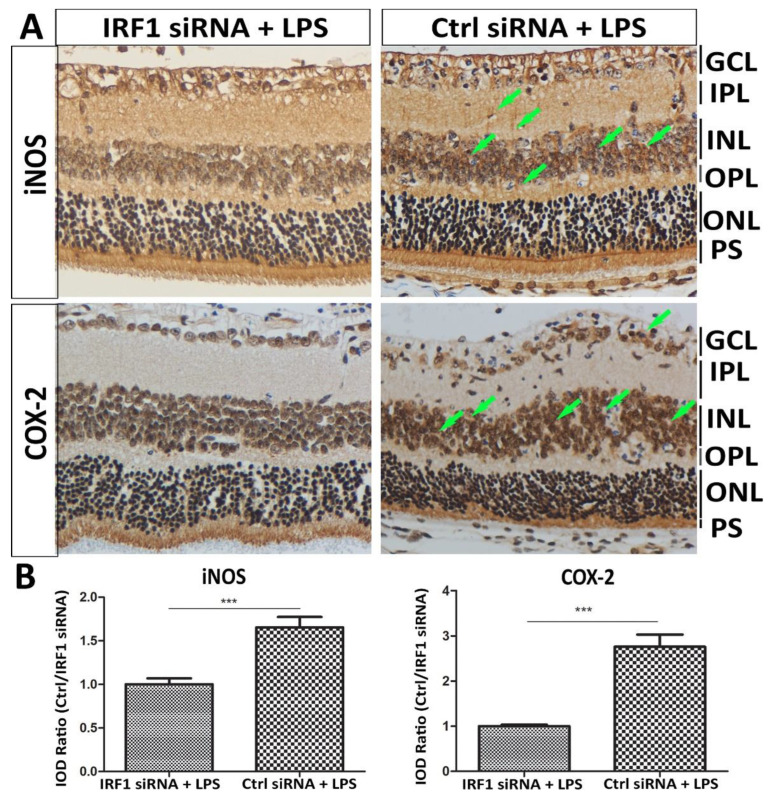
IRF1 knockdown suppresses retinal inflammation induced by LPS in mice. The mice were treated with IRF1-siRNA + LPS and Ctrl siRNA + LPS via intravitreal injection. DAB-IHC staining showed the protein expression levels of inflammatory markers iNOS and COX2. The staining signal intensity indicates the protein expression level (arrows), which was higher in the Müller cellular processes and the inner nuclear layer of the Ctrl siRNA + LPS than the IRF1-siRNA + LPS. (**A**) The representative images are shown. Hematoxylin stained the nuclei. (**B**) The quantification results of IHC staining signals were determined by Image J software-IHC Image Analysis Toolbox. The results are expressed as the integrated optical density (IOD) ratio of Ctrl siRNA + LPS vs. IRF1-siRNA + LPS. *** *p* < 0.001. n = 9 areas of interest from 3 independent biological samples. NFL: Nerve fiber layer. GCL: Ganglion cell layer. IPL: Inner plexiform layer. INL: Inner nuclear layer. OPL: outer plexiform layer. ONL: outer nuclear layer. PS: photoreceptor segment.

The effect of IRF1 siRNA on retinal inflammation was assessed using the expression levels of two inflammatory factors (iNOS and COX-2). Immunoreactivity and quantification indicated that their protein levels were lower in the retina treated with IRF1-siRNA + LPS than the control siRNA + LPS, especially at the inner nuclear layer (INL) and/or Müller cells (Figure 8).

## 3. Discussion

The present study provides new experimental evidence for the IRF1-mediated transcription regulation of microglial activation and retinal inflammation. IRF1-regulated microglial polarization is characteristic of pro-inflammatory gene expression upregulation induced by M1 stimulation (LPS, TNFα, and IFNγ) and anti-inflammatory gene expression downregulation by M2 stimulation (IL-4 and IL-13). The new IRF1-KO BV2 microglia cell line (BV2^ΔIRF1^) created with the CRISPR-Cas9 gene editing technique acts as an attractive and powerful tool to define the role and mechanisms of IRF1 in regulating microglial activation and function. Although IRF1-KO moderately modifies microglial phenotypes, such as deferred microglial cell proliferation rate, reduced phagocytosis function, and migration ability under non-stimulated or steady-state conditions, IRF1-KO drastically changes the expression profiles of pro- and anti-inflammatory genes upon activation or polarization. Microglial-conditioned media (MCM) from the activated BV2^ΔIRF1^ cell cultures caused cellular toxicity and death to retinal cells markedly less than MCM from the WT cell cultures, indicating that IRF1 regulates paracrine mechanisms from microglia to retinal cells. Moreover, IRF1 knockdown by siRNA also robustly limited microglial activation and retinal inflammation in mice. These findings suggest a new potential therapeutic strategy to control dysregulated microglial function and inflammatory retinal diseases by modulating IRF1-mediated microglial polarization or activation states.

IRF1 is the first IRF family member discovered in 1988 and has multiple functions in various diseases [16]. It has been well-characterized that IRF1 is involved in the immune response to viral and bacterial pathogen infection [25]. For example, IRF1 is crucial for host defense against *Mycobacterium bovis* and *M. tuberculosis* by regulating iNOS expression and NO production [26,27]. IRF1 is associated with a variety of cancer progressions. For instance, IRF1 and IRF2 interact synergistically to suppress neuroblastoma by regulating apoptotic caspase-7/8 and MHC-I gene expression [28,29,30]. IRF1 interacts with STATs and NF-κB to regulate vascular cell functions in vascular injury disorders [31]. IRF1, together with other IRF members (e.g., IRF2, IRF4, and IRF8), contributes to the inflammatory process by regulating macrophage M1 polarization and pro-inflammatory gene expressions, such as COX2 and IL-1β, upon activation of INFγ and TLR signaling pathways [19,32]. IRF1 is also implicated in neuroinflammation by regulating the microglial transition from homeostatic to the reactive state by regulating disease-associated microglia (DAM)-related gene expression [20]. IRF1-regulated reactive microglia mediate oligodendrocyte and neuronal cell death in multiple sclerosis, brain injury, and degenerative diseases [33,34,35]. Our study found that IRF1 is upregulated in activated retinal microglia and mediates neuroinflammation in the retina. Moreover, the IRF1-mutant BV2^ΔIRF1^ cells express increased M1 microglial marker genes, such as COX2, IL-6, and iNOS, but decreased M2 marker genes, such as Arg1, CD206, and TGFβ, under the polarization conditions by LPS, TNFα/IFNγ, and IL-4/-13. These data indicate that IRF1 may regulate microglial activation states in retinal diseases. 

iNOS (or NOS2) was the first characterized gene to suggest that IRF1 directly regulates macrophages by interacting with its promoters [36,37]. Subsequent studies revealed that the IRF1–iNOS regulatory axis is conserved in various cell types, such as microglia [38], retinal pigment epithelium (RPE) [39], and vascular cells [40], and mediates pathophysiological events under various conditions, such as ischemic-reperfusion injury [41]. We have obtained new evidence that IRF1 binds with the promoter of the iNOS gene and directly regulates its gene expression, thus resulting in escalated NO levels in the microglial culture supernatant. The free radical NO accumulation, resulting from the activated microglial IRF1–iNOS axis, was hypothesized to cause toxicity and death to retinal cells and contributes to retinal inflammation. IRF1 knockdown experiments with siRNA in mice provided further in vivo evidence that IRF1 can be targeted to alleviate microglial activation and retinal inflammation. IRF1 was reported to regulate many other downstream immune response gene expressions of INF and TLR signaling pathways. However, it is unknown whether IRF1 directly regulates those genes in microglia, for example, by interacting with the cis-regulatory elements in the DNA promoter and enhancer region. The ChIP-sequencing, luciferase activity, and electrophoresis mobility shift assay (EMSA) can comprehensively characterize the IRF1-regulated genes in microglia. The outcomes are expected to give new insights into the transcriptional regulation of microglial activation and identify new potential cis-regulatory elements that control microglia-mediated neuroinflammation.

Besides IRF1, several other IRF family members were reported to be involved in microglial activation and retinal function. IRF2 acts as an antagonist that negatively regulates IRF1 activity, likely by competing with the cis-regulatory binding sites in the promoter of target genes. IRF1 is required for IRF8-regulated IL-1β expression in activated microglia. IRF8 was recently reported to play a critical role in retinal microglial homeostasis in steady state and microglial reactivation in laser injury, which negatively impacts choroidal neovascularization (CNV), leading to reduced CNV size [42]. IRF3 is critical in regulating retinal senescence during aging, as old IRF3^-/-^ mice showed retinal abnormalities in retinal structure and function compared with the young IRF3^-/-^ mice [43]. IRF5 mediates LPS-induced microglia activation and neuroinflammation in the CNS [44]. The IRF5–IRF4 regulatory axis is a determinant of microglial activation in the M1 state vs. M2 state, regulating neuroinflammation following the ischemic cerebral stroke model [45]. These studies demonstrate that IRF family members can work alone and/or in combination to regulate microglial activation and neuroinflammation in the CNS (brain and retina). 

Several questions would be interesting to address in future prospective studies. Firstly, which retinal diseases in which IRF1 is implicated are needed to be clarified? As an initial step, experimental models of retinal diseases created in the IRF1-KO mice (#002762, Jackson Labs, Bar Harbor, ME, USA) and the WT controls can be adapted to elucidate IRF1’s involvement in pathological processes of retinopathies. Secondly, whether the dysregulated IRF1–iNOS signaling axis is the cause of retinal cell death and pathogenesis is inconclusive. Whether and what other pro-inflammatory factors produced by reactive microglia contribute to the paracrine mechanisms from retinal microglia to photoreceptors and neurons is also unknown. Thirdly, as mentioned above, what other downstream genes of INFγ and TLR signaling pathways that IRF1 regulates in microglial cells will be further characterized. What microglial genes do IRF1 directly regulate by interacting with their promoters, indirectly by synergically interacting with other transcription factors, or through secondary effects? Fourthly, whether and how IRF1 regulates microglial polarization or activation states (e.g., pro-inflammatory M1 and anti-inflammatory M2) under specific stimulation conditions and phenotypic/functional heterogeneities during diseases is highly desired to be elucidated since this question represents a critical knowledge gap in the microglia research field. Lastly, whether and how IRF1 can be targeted to delay or prevent microglia-mediated retinal inflammatory diseases would be interesting to explore further. Addressing these questions can advance our understanding of the mechanisms underlying microglial activation and guide the design of therapeutic strategies to limit retinal inflammation. 

In summary, IRF1 is a critical transcription factor that mediates gene transcription in microglia. Microglial activation regulated by IRF1 represents an important paracrine mechanism regulating retinal cell death and retinal inflammation. Gene silencing and pharmacological approaches targeting IRF1 are potential strategies to prevent or delay inflammatory and degenerative retinal diseases. 

## 4. Materials and Methods

### 4.1. Experimental Animals

All animal experiments were approved by the Institutional Animal Care and Use Committee of the University of Missouri (Protocol number: 36223) and conducted in compliance with the “Statement for the Use of Animals in Ophthalmic and Vision Research” of the Association for Research in Vision and Ophthalmology (ARVO). All mice were housed in pathogen-free animal facilities in the Medical Sciences Building at the University of Missouri (MU), Columbia, MO, USA and were fed normal chow diets and provided with water ad libitum.

### 4.2. Intravitreal Injection of LPS and siRNA in Mice

Mice were anesthetized with the intraperitoneal (IP) injection of ketamine (100 mg/kg) and xylazine (10 mg/kg). Intravitreal injections of IRF1 siRNA (1 μL, 1 µg/µL) and LPS (1 µL, 125 ng/µL) were performed at the site of ~1 mm behind the white limbus ring with the 30 G needle administrated by Hamilton syringe (10 µL). IRF1 siRNA was injected twice on day 0 and day 3; LPS was done once on day 5. On day 6, the mice were euthanized with excessive CO_2_ for sample collection and further analyses. 

### 4.3. Retinal Flat Mounts and Immunofluorescent Stain

The eyeballs were enucleated and fixed with 4% PFA at room temperature for 1 h. Anterior segment and vitreous tissues were dissected away under a dissection microscope to make eyecups. The retinas were gently separated from the RPE–choroid–sclera complexes and four relaxing radial incisions were made. The prepared retinas were blocked and permeabilized with 1% Triton-×100 and 5% goat serum for 1 h, followed by incubation with primary antibodies (see Table 1 at 4 °C for 2 days and secondary antibodies coagulated with fluorescent dyes (e.g., Alexa Fluor 488, Alexa Fluor 594, and Cy5) at 4 °C for 1 day. The nuclei were counterstained with DAPI. The stained retinas were flat—mounted on slides with the ProLong Diamond antifade reagent for confocal microscopy imaging analysis. Control samples incubated with a blocking buffer (the primary antibody was omitted) followed by secondary antibody incubation were used to appropriate confocal settings for non-specific backgrounds.

### 4.4. Cell Cultures and Treatments

Primary retinal microglia were dissected from the ~p10 C57BL6/J mouse pups and cultured according to previously described procedures [10]. Briefly, about twenty retinae were pooled and incubated with papain proteinase. The digested retina was triturated to dissociate the tissues and make cell suspensions. After the cell debris and large cell clumps were removed by filtration through a 70 μm cell strainer, the resultant retinal cell suspensions were cultured with DMEM/F12 + 10%FBS + 1% P/S + 10% LADMAC culture medium (or 10 μg/mL M-CSF). A total of 80% confluent cell cultures were treated with 100 ng/mL LPS and PBS (control) for 24 h. Rat retinal precursor cells R28 (EUR201, kerafast) were cultured with a complete DMEM+ medium (https://www.kerafast.com/item/717/retinal-cell-line-r28) (accessed on 23 November 2022) [46]. Mouse cone photoreceptor-like cells (661 W) [47,48] and human ARPE-19 cells (CRL-2302, ATCC) were cultured with the complete DMEM/F12 medium with high glucose, 10% FBS, and 1% P/S antibiotics. A total of 80% confluent cells were treated with the microglia-culture media (CM) for 24 h, both for microglia without stimuli or under stimulation conditions. 

### 4.5. IRF1 Knockdown (KD) and Overexpression (OE) in BV2 Microglial Cells

Microglial BV2 cells (CRL2469, ATCC) were seeded in a 6-well plate at a density of 6 × 10^5^ cells/well and cultured with DMEM supplemented with 10% FBS, 1% P/S, and 10% LADMAC culture media. A total of 80% confluent BV2 cells were transfected with IRF1 siRNA (scramble siRNA as control) or IRF1 OE plasmid (pCDH–CMV–IRF1, pCDH–CMV–RFP as controls) via Lipofectamine (Invitrogen, Carlsbad, CA, USA) and Opti-MEM medium (Gibco, Gaithersburg, MD, USA). After 48 h of transfection, cells were harvested for qPCR and WB analyses.

### 4.6. IRF1 Knockout (KO) from BV2 Cells with the CRISPR/Cas9 Method

The CRISPR/Cas9 genome-editing experiments were performed according to the procedures described in the literature [49]. A small guide (sg)RNA was designed based on the IRF1 gene sequence. The sgRNA oligo-nucleotide sequence (Table 2) was designed, synthesized, and cloned into the lentiCRISPR v2 plasmid (#52961, Addgene) to construct the lenti-guide-puro-sgIRF1 (sgIRF1) recombinant plasmid. The lentiviral particles were assembled and produced in HEK293T cells through the co-transfection of the sgIRF1 vector with the 2 packaging plasmids (psPAX2 and pMD2.G) using a PEI 40K reagent. The lentivirus was collected from the culture medium at 48 h post-transfection and filtered through a 0.2 μm sterile filter. The BV2 microglial cells were infected with harvested recombinant lentiviruses and maintained in a fresh medium with 10 μM/mL polybrene for 24 h. Lentiviral-transduced BV2 cells were selected in 10 μg/mL puromycin for 5 days. The survival cells from the antibiotic selection were diluted into single colonies and further cultivated. The expanded single-cell colonies confirmed with IRF1 gene mutation by DNA sequence and protein absence by WB analysis were designated BV2^ΔIRF1^ cells.

### 4.7. Cell Live and Death Assays 

Retinal cells, including murine cone-like 661 w cells, rat retinal neuron (R28), and human ARPE-19 cells, were cultured until ~80 confluent (see above). The microglia–CM were supplemented into the culture media and incubated for 24 h. The live cells were visualized with Calcein-AM (viability) dye. The dead cells were shown with propidium iodide (P3566; Thermo Fisher; 1:3000 dilution).

### 4.8. Cell Migration Assay

BV2 microglial cell migration ability was evaluated with an in-vitro cell scratch assay as performed previously [2]. In brief, the semi-confluent BV-2 microglial cells were induced with a scratch along the diameter of the well with a sterile razor. The detached cells were then removed by washing with PBS, supplied with a fresh complete culture medium, and treated with 100 ng/mL of LPS. PBS-treated wells were used as controls. Twenty-four hours after treatment, the cells were imaged with light microscopy using a phase contrast lens; the repopulation of the scratched area and the distance from the starting point to where the furthest cell migrated were measured and quantified. Three replicates were applied for the treatment condition, three images were taken from each well, and the quantification results were averaged.

### 4.9. Microglia Phagocytosis of Zymosan Bioparticles

BV2 microglia cells expressing different phenotypes (BV2 WT, BV2^ΔIRF1^, and BV2^IRF1-OE^) were seeded (25,000 cell/well) into the 96-well plate coated with L-poly-lysine (12.5 ug/mL, 2 h, #P0899, Sigma, Sofia, Bulgaria) and then cultured for 24 h before treatment. The cells were treated with LPS and PBS (as control) for 4h and then incubated with pHrodo™ Green Zymosan Bioparticles™ (100 uL, 0.5 mg/mL, #P35365, Thermo Fisher, Waltham, MA, USA) for 1.5 h. After washing with PBS, the cells were fixed for confocal microscopy and ImageJ analysis to quantify the pixel intensity of Zymosan bioparticles within the cells. Briefly, the fluorescent microscopy images were converted into gray-model images. The pixel number of Zymosan bioparticles was analyzed and determined by the ImageJ software. The pixel intensity quantification was defined as the quotient of the total pixel number divided by the cell number. The averaged pixel intensity per cell represents the phagocytosis activity of microglial cells. 

### 4.10. Flow Cytometry

10^5^ IRF1-knockout BV2 (BV2^ΔIRF1^, or BV2-IRF1 KO) and wild-type (WT) BV2 cells were seeded into the 6-well plates (6 replicates each cell) and were grown for 24 h. BrdU (10 µM) was added into the culture media and incubated at 37 °C for 2 h. The cells were washed with PBS, harvested with trypsin, had debris removed by centrifugation, and fixed with 4% PFA. The prepared cell suspensions were incubated with the anti-BrdU antibody conjugated with the Alex Fluor 488 dye and then detected with the BD FACSCelesta Flow Cytometer (the cells without BrdU were used as negative controls to set up the parameters). The data were analyzed with FlowJo Software (Version 3.0, FlowJo LLC/Becton Dickinson, Ashland, OR, USA). For the flow cytometry photoreceptor live/dead assay, 10^5^ cone-like 661 W cells were seeded into the 12-well plates and grown for 24 h. The conditioned culture media (CM) were used with the microglia under four conditions, BV2 WT-PBS, BV2 WT-TNF, BV2-IRF1 KO-PBS, and BV2-IRF1 KO-TNF (3 replicates/condition) and microglia were added into the 661 W culture media and incubated for 12 h. The cells were processed and stained with Calcein-AM/Propidium iodide (PI) for the flow cytometry assay as described above (the cells without Calcein/PI dye were used as negative controls). 

### 4.11. Real-Time Quantification PCR

mRNA expression was quantified with real-time quantitative PCR (qPCR) analysis. Total RNA was isolated with the RNeasy Mini kit (Qiagen, Germantown, MD, USA), and mRNA transcripts were reverse-transcribed to cDNA in a SimpliAmp Thermal Cycler (Thermo Fisher, Waltham, MA, USA). cDNA and primers (Table 2) were mixed with SYBR Green dye for PCR reaction with the Quant Studio-3 qPCR system (Thermo Fisher, Waltham, MA, USA), which also detects and captures the fluorescent signals during PCR reactions in real time. The fluorescence signals of the housekeeping gene GAPDH were normalized to those generated from target genes. The normalized signal intensity was used to calculate relative changes between control and treatment groups using the 2^−ΔΔCT^ algorithm. Each group included three biological and three technical replicates for statistical analysis.

### 4.12. Western Blots and Densitometry Analysis

Western blots (WB) were performed with the experimental procedures as described previously [23]. The cells were washed with cold PBS three times, detached with a cell scraper, and collected by centrifugation (500× *g*, 5 min). Cell pellets were dissolved with cold radioimmunoprecipitation assay buffer (RIPA) containing protease inhibitors (Cat#: S8830, Sigma, Sofia, Bulgaria). After incubation in ice for 10 min and centrifugation at 12,000× *g* for 10 min, the protein concentration was determined with the BCA assay. A total of 30–50 μg of protein per lane was separated by SDS-PAGE gel (4–20% gradient) electrophoresis and transferred to nitrocellulose membranes (0.45 μm pore size). The protein blots were blocked with 5% BSA (or non-fat milk) for 1 h, followed by incubation with the primary antibodies (Table 1) overnight at 4 °C. Protein blots were washed with PBS-T buffer and incubated with an HRP-conjugated secondary antibody (1:1000) for 1 h. The chemiluminescent signals were developed with the SuperSignal West Pico kit (Thermo Fisher, Waltham, MA, USA) and captured with the ImageQuant LAS 500 instrument (GE Healthcare, Chicago, IL, USA). The pixel intensity was determined with the densitometric analysis of ImageJ software (NIH, Bethesda, MD, USA). All the quantification results were averaged from three protein blots and expressed as the mean ratio of the values (target protein/housekeeping protein) ± standard deviation (SD).

### 4.13. Immunohistochemistry and Immunofluorescence

Immunohistochemistry (IHC) and immunofluorescence (IF) were performed according to previously described procedures [23,50]. Eyeballs were fixed with fixative solutions (10% formalin and 4% PFA), washed, and stored in PBS until use. The fixed specimens were embedded with paraffin or cryopreserved with OCT compounds for sectioning. The sections were permeabilized by incubation in 0.05% Triton X-100 for 10 min and blocked with 2.5% normal goat serum for 1 h at room temperature (RT). The samples were incubated with primary antibodies (Table 1) and appropriate secondary antibodies coagulated with chemical enzymes or fluorescent dye. For IHC, horseradish peroxidase (HRP)-conjugated secondary antibody (1:1000; Bio-Rad, Hercules, CA, USA) was applied and incubated at room temperature for 1 h. The staining signals were visualized by incubation with DAB substrate (34002; Thermo Fisher, Waltham, MA, USA) for 7 min. For IF staining, the signals were visualized by the secondary antibodies coagulated with Alexa Fluor 488, Alexa Fluor 594, Alexa Fluor 647, or Cyanine5. The cell nuclei were visualized by incubation with 4′,6-diamidino-2-phenylindole (DAPI). The slides were mounted with ProLong Diamond antifade reagent (Thermo Fisher, Waltham, MA, USA). The images were taken and analyzed with light, fluorescent, or confocal microscopy. 

### 4.14. Statistical Analysis

Statistical analyses were performed with GraphPad Prism software. The Student’s *t*-test was used to compare the two groups, and a one-way analysis of variance with Tukey’s multiple comparisons was used to compare multiple groups. A *p*-Value of <0.05 was considered statistically significant. 

## Figures and Tables

**Table 1:** Antibody information.

Target Protein & Secondary Antibody (2 Ab)	Produced by	Catalog Number	Host	WB Dilution	IF Dilution
IRF1	CST	#8478	rabbit	1:1000	-
inos	abcam	ab283655	rabbit	-	1:100
GAPDH	arigo	ARG10112	mouse	1:1000	-
actin	arigo	ARG62346	mouse	1:1000	-
Iba1	wako	019-19741	rabbit	-	1:500
GFAP	Thermo	13-0300	Rat	-	1:100
DAPI	Thermo	C0060		-	1:5000
Goat anti-Mouse IgG antibody (HRP)	arigo	ARG65350	Goat	1:5000	-
Goat anti-Rabbit IgG antibody(HRP)	arigo	ARG65351	Goat	1:5000	-
Goat anti-Mouse IgG 2 Ab, Alexa Fluor 488	thermo	A-11001	Goat	-	1:500
Donkey anti-Rat IgG 2 Ab, Alexa Fluor Plus 488	thermo	A48269	Donkey	-	1:500
Goat anti-Rabbit IgG 2 Ab, Cyanine3	thermo	A10520	Goat	-	1:500

**Table 2:** Nucleotide sequence.

Oligo Name	Sequence
IRF1-siRNA-01	GCACCACTGATCTGTATAA
IRF1-siRNA-02	CCAAGACATGGAAGGCAAA
IRF1-siRNA-03	GGACATTGGGATAGGCATA
IRF1-sgDNA	CCATGCCAATCACTCGAATG
CCL5-Forward	TGCCCACGTCAAGGAGTATT
CCL5-Reverse	GCGGTTCCTTCGAGTGACA
iNOS-Forward	GGTGAAGGGACTGAGCTGTT
iNOS-Reverse	CGTTCTCCGTTCTCTTGCAGT
COX2-Forward	GCGAGCTAAGAGCTTCAGGA
COX2-Reverse	TCATACATTCCCCACGGTTT
CCL2-Forward	TTAAAAACCTGGATCGGAACCAA
CCL2-Reverse	GCATTAGCTTCAGATTTACGGGT
IL-6-Forward	TAGTCCTTCCTACCCCAATTTCC
IL-6-Reverse	TTGGTCCTTAGCCACTCCTTC
CD47-Forward	GGTGGGAAACTACACTTGCGAAG
CD47-Reverse	CTCCTCGTAAGAACAGGCTGATC
Arg1-Forward	ACTGGAACCCAGAGAGAGCA
Arg1-Reverse	ACAGACCGTGGGTTCTTCAC
CD206-Forward	TTCAGCTATTGGACGCGAGG
CD206-Reverse	GAATCTGACACCCAGCGGAA
TGF-b-Forward	AGCCCGAAGCGGACTACTAT
TGF-b-Reverse	TCCACATGTTGCTCCACACT
Actin-Forward	AGGCGGACTGTTACTGAGC
Actin-Reverse	CGCCTTCACCGTTCCAGTT

## Data Availability

All data generated and analyzed in the current study are included in this published article and its supplementary information.

## Supplementary Materials

The supporting information can be downloaded at: https://www.mdpi.com/article/10.3390/ijms232314664/s1.
