# Supplementary material for: The Role of Interferon Regulatory Factor 1 in Regulating Microglial Activation and Retinal Inflammation
Source: Int J Mol Sci. 2022 Nov 24;23(23):14664. doi: 10.3390/ijms232314664 (PMC9739975; doi:10.3390/ijms232314664)
Supplement: Supplementary file 1 [file ijms-23-14664-s001.zip › ijms-1993691-supplementary.pdf]

Supplementary Materials

The Role of Interferon Regulatory Factor 1 in Regulating Microglial Activation and Retinal Inflammation

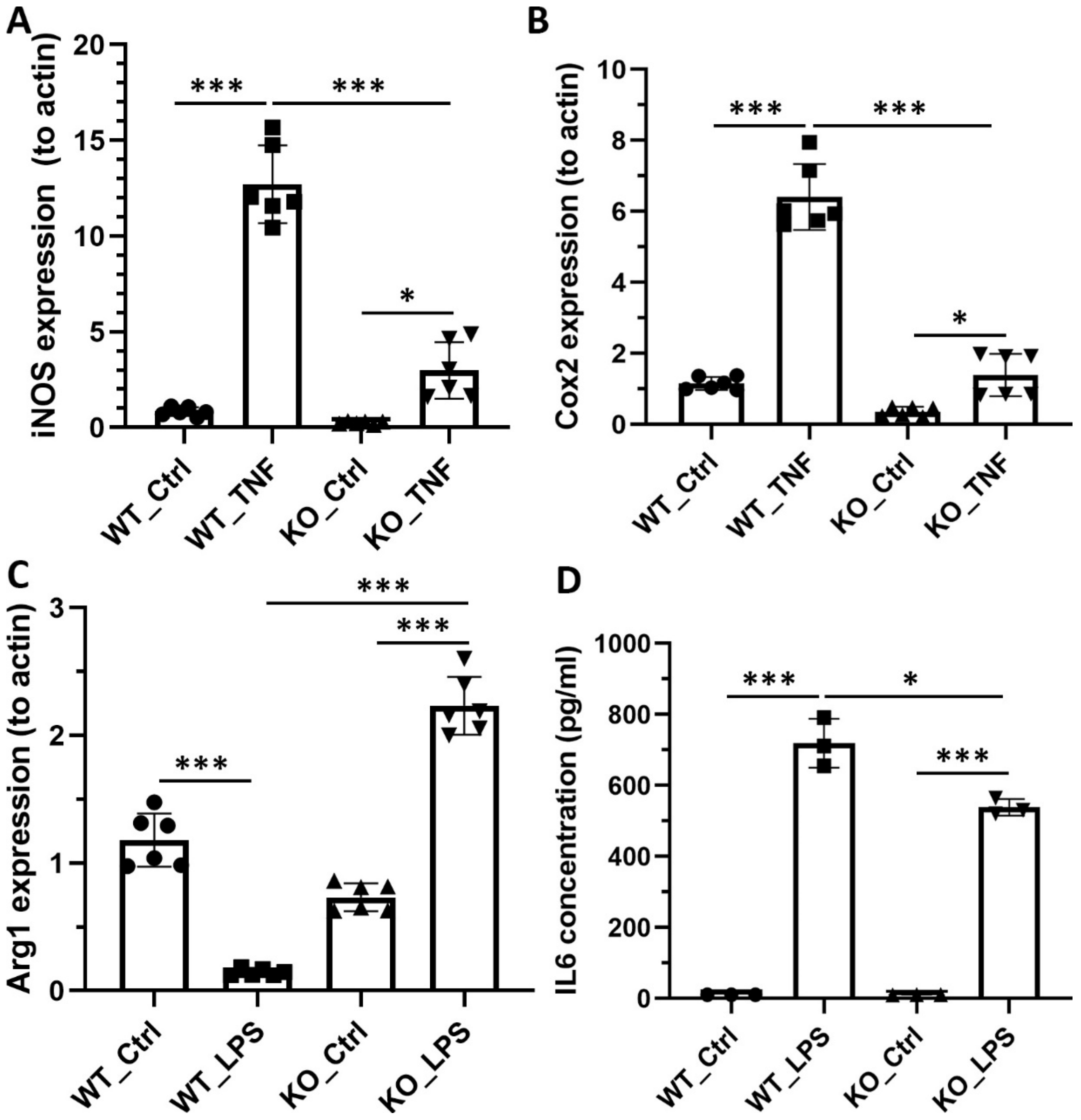

Figure S1. **IRF1 KO promotes gene expression profiles of the M2 microglial activation state.** The IRF1 KO BV2 cells (BV2$^{\Delta IRF1}$) and wild-type control cell cultures were treated with LPS or TNF (PBS as control) for 24h. The qPCR results for mRNA expression of iNOS (A), COX-2 (B), and Arg1 (C). (D) The protein concentration of IL-6 by ELISA assay. The replicate numbers = 6 for qPCR and 3 for ELISA. * P < 0.05; *** P < 0.001.

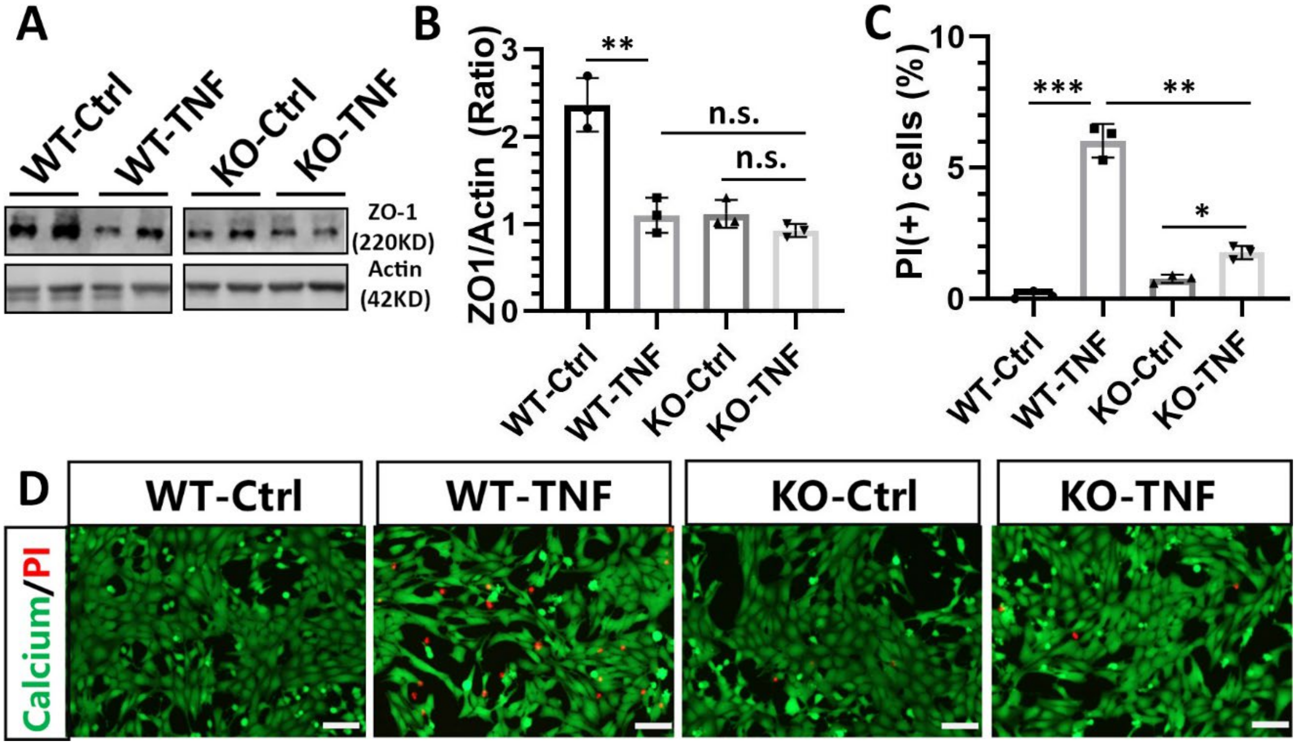

Figure S2. IRF1 deletion reduces microglial cytotoxicity to RPE and retinal neurons. (A) ZO-1 western blotting images for human ARPE-19 cells treated with the conditioned culture media (CM) from the BV2 wild type (WT) cells under PBS condition (WT-Ctrl), the BV2 WT cells under TNF condition (20ng/ml, 24h, WT-TNF), the IRF1-KO BV2 ($BV2^{\Delta IRF1}$) cells under PBS condition (KO-Ctrl), and the $BV2^{\Delta IRF1}$ cells under TNF condition (WT-TNF). (B) Densitometry quantification results of the western blots. (C) Cell death quantification of the calcium AM and PI stained images for rat R28 retinal neuronal precursor cells treated with the CM from BV2 microglia under the four conditions as above. (D) The representative images showing the Calcium AM (green) and PI (red) stained R28 cells. The replicate numbers = 3. * $P < 0.05$; ** $P < 0.01$; *** $P < 0.001$ (D).
